# Supplementary material for: Non-Canonical Activin A Signaling Stimulates Context-Dependent and Cellular-Specific Outcomes in CRC to Promote Tumor Cell Migration and Immune Tolerance
Source: Cancers (Basel). 2023 May 31;15(11):3003. doi: 10.3390/cancers15113003 (PMC10252122; doi:10.3390/cancers15113003)
Supplement: Supplementary file 1 [file cancers-15-03003-s001.zip › cancers-2358682-supplementary.pdf]

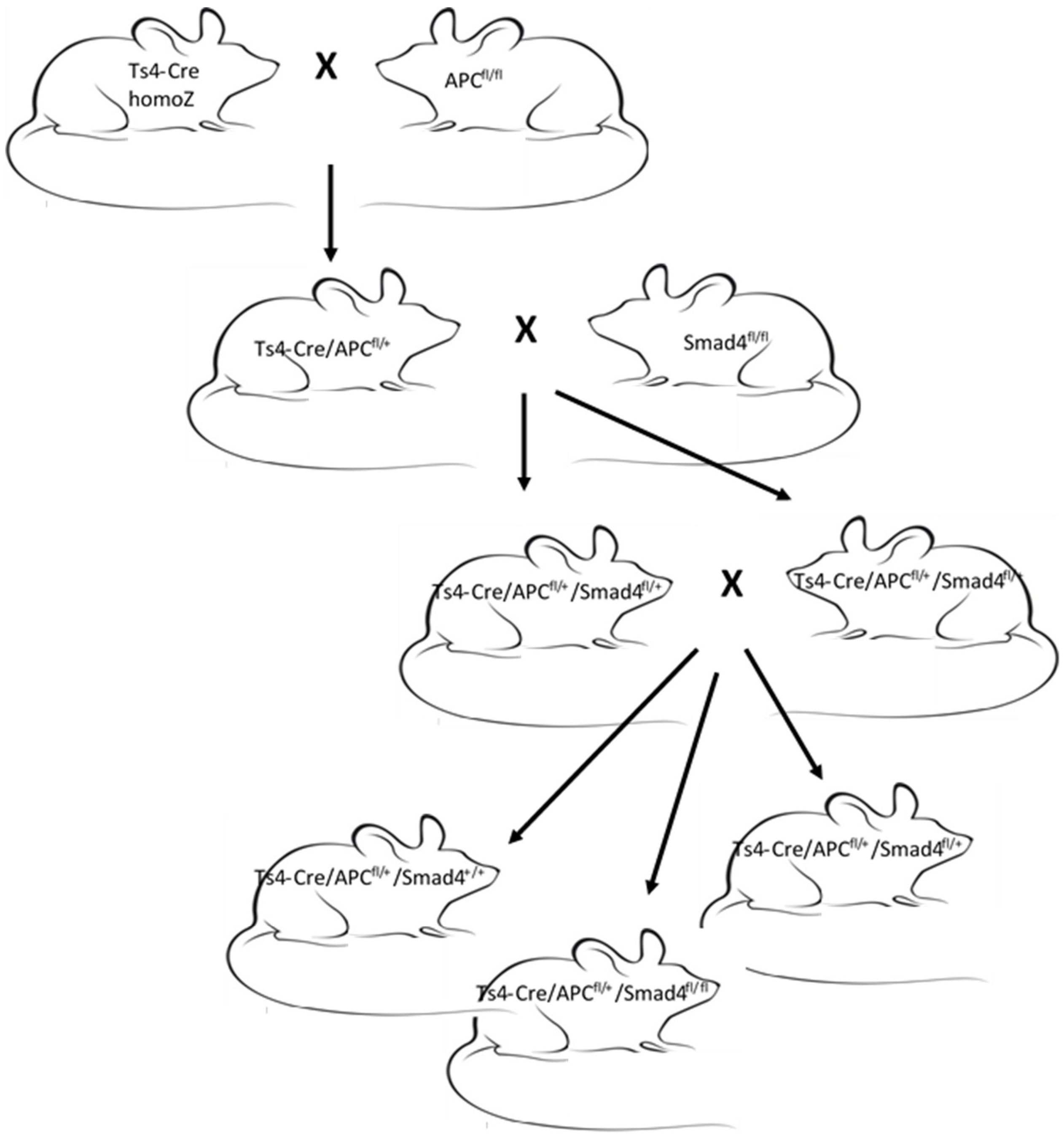

Primers:

<u>**APC$^{\Delta 468}$**</u>
Fapc up 5'- GTA TTC TCA GTC TTA GCG TTC T -3'
Fapc low 5' - TTA ACA AGG GCA AAA GGA AAC - 3'
<u>**Ts4-Cre**</u>
Ts4Cre 5' 5'- TGA CCG TAC ACC AAA ATT TG -3'
Ts4Cre 3' 3'- ATT GCC CCT GTT TCA CTA TC -3'

Reaction Components:

Reaction Component	Conc	Volumes
DNA		1 ul
d H_2O		7 ul
2X GoTaq Mastermix	10x	10 ul
Primer 1	10 uM	2 ul
Primer 2	10 uM	2 ul
**total**		**20 ul**

Cycling Conditions:

APC

Step	Temperature	Time	Note
**1**	94 °C	5 min	
**2**	94 °C	45 sec	
**3**	60 °C	45 sec	-1 °C per cycle
**4**	72 °C	1 min	go to step 2 , 6 times
**5**	95 °C	30 sec	
**6**	54 °C	30 sec	
**7**	72 °C	30 sec	go to step 5 , 34 times
**8**	72 °C	7 min	
**9**	4 °C	∞	

Ts4-Cre

Step	Temperature	Time	Note
**1**	94 °C	5 min	
**2**	94 °C	45 sec	
**3**	60 °C	45 sec	-1 °C per cycle
**4**	72 °C	1 min	go to step 2 , 6 times
**5**	95 °C	45 sec	
**6**	54 °C	45 sec	
**7**	72 °C	1 min	go to step 5 , 31 times
**8**	72 °C	7 min	
**9**	4 °C	∞	

- Separated by gel electrophoresis on a **2.0%** agarose gel
- Band size:
  APC: +/+ 115bp (APC *wt*), +/- 115bp/ 135bp (het), -/- 135 (APC exons 11+12 loxp)
  Ts4-Cre 1000bp (positive; no band negative)

Sample Agarose Pictures:

<u>**APC**</u>

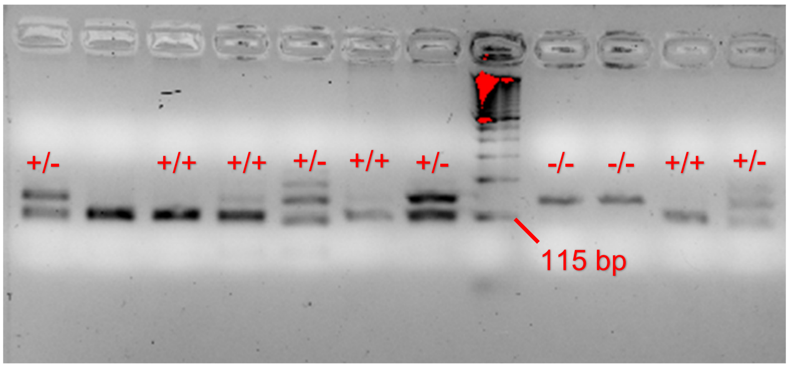

<u>**Ts4-Cre**</u>

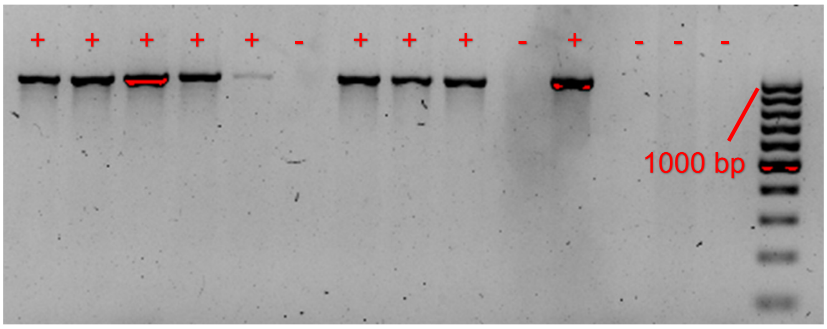

Supplemental Figure S1: Schematic of the genetic approach used to generate mice that develop polyps in the large intestine and lack the SMAD4 protein in the epithelial cells of the large intestine. PCR primer and cycle information and sample images from genotyping are also included.

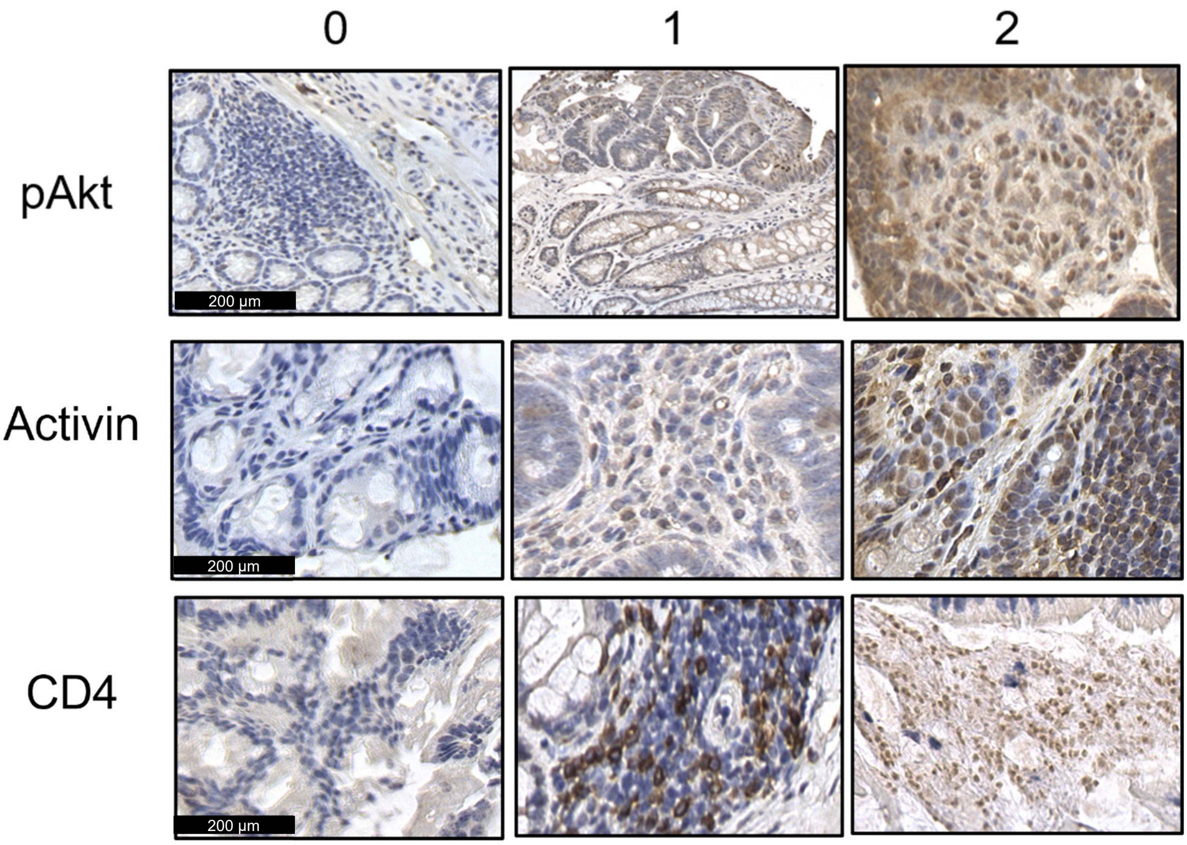

Supplemental Figure S2: Representative images of low (0, left column) medium (1, middle column) and high (2, right column) scores for pAkt (top row), activin (middle row), and CD4 (bottom row) from the colon of mice in the Ts4Cre/cAPC^{flox}/Smad4^{flox} experiments.

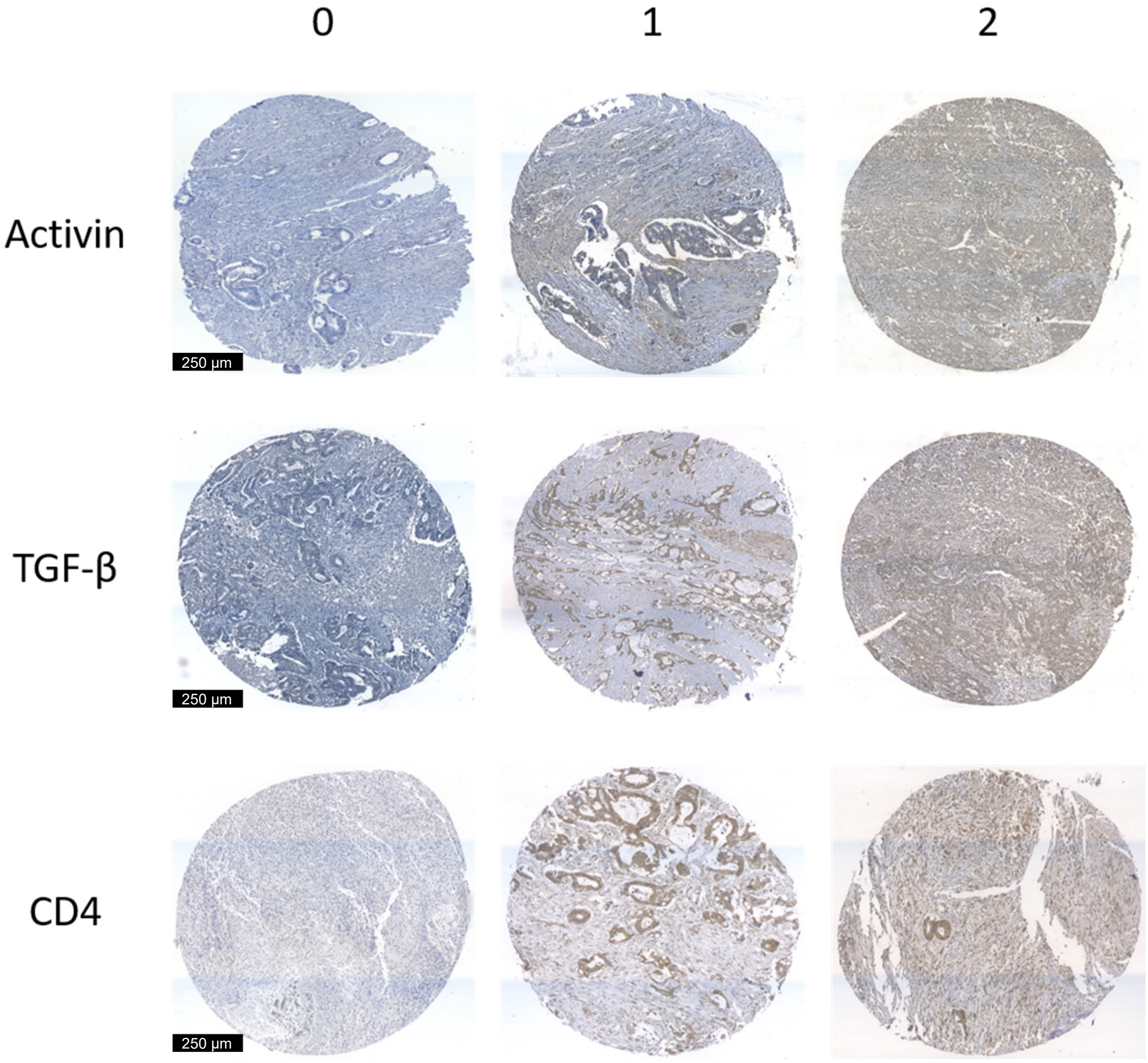

Supplemental Figure S3: Representative images of human CRC IHC images for low (0, left column), medium (1, middle column), and high (2, right column) scores for activin (top row), TGF-β (middle row), and CD4 (bottom row).

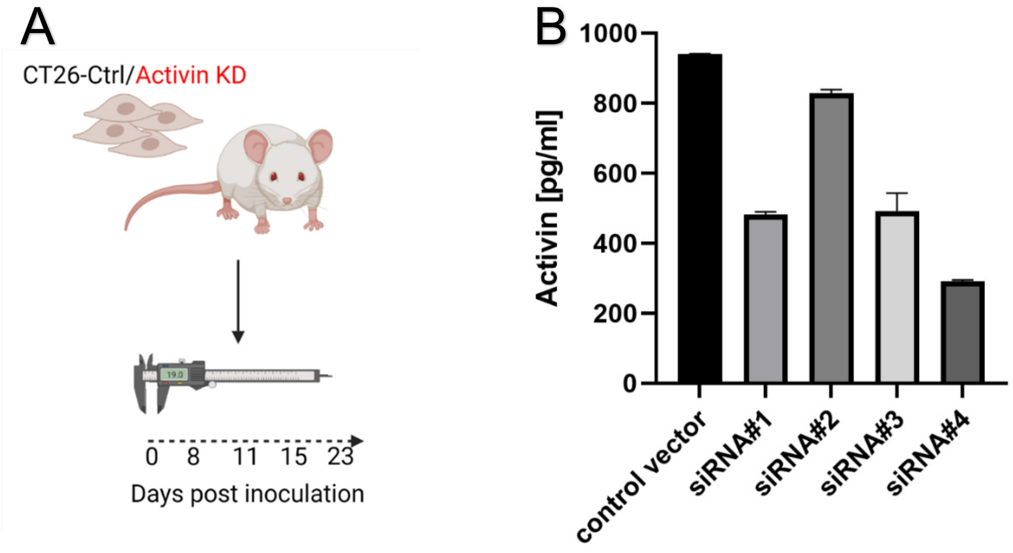

Supplemental Figure S4: (A) Schematic displaying the approach employed for the activin knockdown (KD) *in vivo* subcutaneous tumor model. Created with BioRender.com **(B)** ELISA data collected from CT26 cells treated with control vector siRNA or one of four siRNA variants which all targeted *INHBA*. Given the greatest decrease in Activin production in response to siRNA#4, this was the vector used in the mouse experiments.

Supplemental Table S1: List of the 57 quantitative markers employed in the DSP experiments including both positive and negative controls which were used for normalization.

Immune Cell Profiling		Immune Cell Typing	
PD-1	CD45	FOXP3	FAP-alpha
CTLA4	CD56	CD45RO	CD14
HLA-DR	CD8	CD34	CD163
Ki-67	CD68	CD66b	
Beta-2-macroglobulin	GZMB	**Immune Cell Phenotyping**	
CD11c	PD-L1	CD127	PD-L2
CD20	PanCk	CD25	CD40
CD3	SMA	CD80	CD44
CD4	Fibronectin	ICOS	CD27
**MAPK Signaling Pathway**		**PI3K/AKT Signaling Pathway**	
EGFR	Phospho-MEK1	Pan-AKT	Phospho-GSK3A/GSK3B
pan-RAS	Phospho-p38 MAPK	MET	INPP4B
BRAF	Phospho-p44/42 MAPK ERK 1/2	Phospho-AKT1	PLCG1
Phospho-c-RAF	Phospho-p90 RSK	Phospho-Tuberin	Phospho-PRAS40
Phospho-JNK		Phospho-GSK3B	

**Housekeeping Proteins**	Histone H3	S6	GAPDH	**Negative Controls**	Rb IgG	Ms IgG1	Ms IgG2a

Supplemental Table S2: Quantified data from the DSP study which is expressed as counts normalized using the approach described in the methods. Statistical differences between the groups were determined via Linear Mixed Modeling (LMM) with Benjamin-Hochberg correction test.

Protein of Interest	Activin (+) Tumor n = 10	Activin (-) Tumor n = 8	Activin (+) Stroma n = 18	Activin (-) Stroma n = 20
αSMA	96.57 ± 28.61	87.03 ± 16.99	469.06 ± 123.14	646.45 ± 125.132
PanCK	8.89 ± 2.26	5.44 ± 2.07	3.06 ± 1.01	2.21 ± 0.56
CD45	2.35 ± 0.41	3.24 ± 0.73	6.67 ± 1.36	8.46 ± 1.28
CD4	1.17 ± 0.38	0.67 ± 0.16	2.16 ± 0.39	2.02 ± 0.21
CD8	1.04 ± 0.17	0.87 ± 0.18	2.63 ± 0.41	2.00 ± 0.32
CTLA-4	14.49 ± 5.33	7.56 ± 5.44	19.21 ± 5.56	3.66 ± 0.82
FOXP3	0.87 ± 0.28	0.21 ± 0.07	0.77 ± 0.20	0.27 ± 0.05
CD25	0.82 ± 0.27	0.27 ± 0.07	0.95 ± 0.20	0.36 ± 0.08
PD-1	1.97 ± 0.67	0.47 ± 0.13	1.47 ± 0.30	0.99 ± 0.16
HLA-DR	2.47 ± 0.97	2.70 ± 1.31	4.71 ± 1.33	7.17 ± 2.15
CD80	1.07 ± 0.35	0.16 ± 0.06	1.01 ± 0.28	0.32 ± 0.07
CD40	0.91 ± 0.26	0.28 ± 0.15	1.00 ± 0.31	0.46 ± 0.14
PLCG1	0.96 ± 0.23	0.40 ± 0.09	0.87 ± 0.20	0.41 ± 0.06
Phospho-PRAS40	1.47 ± 0.61	0.25 ± 0.07	1.17 ± 0.38	0.34 ± 0.06
Phospho-Tuberin	0.97 ± 0.28	0.22 ± 0.06	0.79 ± 0.19	0.34 ± 0.07

Supplemental Table S3: P-values for each significantly differentially expressed protein found in the DSP analysis. Statistical differences between the groups were determined via Linear Mixed Modeling (LMM) with Benjamin-Hochberg correction test.

Protein of Interest	Activin (+) Tumor vs. Activin (-) Tumor	Activin (+) Stroma vs. Activin (-) Stroma	Activin (+) Tumor vs. Activin (+) Stroma	Activin (-) Tumor vs. Activin (-) Stroma
αSMA	*n.s.* p = 0.881	** p = 0.0046	**** p = 0.00008	**** p = 5.997 x 10^{-7}
PanCK	* p = 0.0186	*n.s.* p = 0.316	**** p = 0.000036	* p = 0.036
CD45	*n.s.* p = 0.182	* p = 0.0283	*** p = 0.00012	*** p = 0.00012
CD4	*n.s.* p = 0.164	*n.s.* p = 0.596	* p = 0.0242	*** p = 0.00023
CD8	*n.s.* p = 0.511	*n.s.* p = 0.304	* p = 0.0138	* p = 0.0227
CTLA-4	** p = 0.0033	*** p = 0.0003	*n.s.* p = 0.651	*n.s.* p = 0.417
FOXP3	** p = 0.00498	* p = 0.0151	*n.s.* p = 0.775	*n.s.* p = 0.375
CD25	* p =0.0286	** p = 0.0093	*n.s.* p = 0.824	*n.s.* p = 0.836
PD-1	* p = 0.0104	*n.s.* p = 0.504	*n.s.* p = 0.668	*n.s.* p = 0.0628
HLA-DR	*n.s.* p = 0.462	** p = 0.00604	*n.s.* p = 0.0552	*** p = 0.00041
CD80	*** p = 0.00032	** p = 0.0027	*n.s.* p = 0.899	*n.s.* p = 0.111
CD40	* p = 0.0131	* p = 0.0231	*n.s.* p = 0.725	*n.s.* p = 0.122
PLCG1	* p = 0.0245	* p = 0.0218	*n.s.* p = 0.576	*n.s.* p = 0.820
Phospho-PRAS40	** p = 0.0045	* p =0.023	*n.s.* p = 0.538	*n.s.* p = 0.357
Phospho-Tuberin	** p = 0.0013	** p = 0.00598	*n.s.* p = 0.663	*n.s.* p = 0.272
